# Characterizing longitudinal change in accelerometry-based moderate-to-vigorous physical activity in the Hispanic Community Health Study/Study of Latinos and the Framingham Heart Study

**DOI:** 10.1186/s12889-023-16442-9

**Published:** 2023-08-24

**Authors:** Yasmin Mossavar-Rahmani, Juan Lin, Stephanie Pan, Rebecca J. Song, Xiaonan Xue, Nicole L. Spartano, Vanessa Xanthakis, Daniela Sotres-Alvarez, David X. Marquez, Martha Daviglus, Jordan A. Carlson, Humberto Parada, Kelly R. Evenson, Ana C. Talavera, Marc Gellman, Krista M. Perreira, Linda C. Gallo, Ramachandran S. Vasan, Robert C. Kaplan

**Affiliations:** 1 Department of Epidemiology & Population Health, Albert Einstein College of Medicine, 1300 Morris Park Avenue, Belfer Bldg, 1312C, Bronx, NY 10461 USA; 2 Section of Preventive Medicine and Epidemiology, Boston University Chobanian and Avedisian School of Medicine, Boston, MA 02118 USA; 3 Department of Epidemiology, Boston University School of Public Health, Boston, MA 02118 USA; 4 Department of Medicine, Boston University Chobanian and Avedisian School of Medicine, Boston, MA 02118 USA; 5 Framingham Heart Study, Framingham, MA 01701 USA; 6 Department of Biostatistics, Boston University School of Public Health, Boston, MA 02118 USA; 7 Department of Biostatistics, Gillings School of Global Public Health, Collaborative Studies Coordinating Center, University of North Carolina, Chapel Hill, NC 27516 USA; 8 Department of Kinesiology & Nutrition, University of Illinois Chicago, Chicago, IL 60612 USA; 9 Institute for Minority Health Research, University of Illinois Chicago, Chicago, IL 60612 USA; 10 Department of Pediatrics, Children’s Mercy Hospital and University of Missouri-Kansas City School of Medicine, Kansas City, MO 64108 USA; 11 Division of Epidemiology & Biostatistics, San Diego State University School of Public Health, San Diego, CA 92182 USA; 12 Department of Epidemiology, Gillings School of Global Public Health, University of North Carolina, Chapel Hill, NC 27599 USA; 13 South Bay Latino Research Center, College of Sciences, San Diego State University, San Diego, CA 92182 USA; 14 Department of Psychology, University of Miami, Coral Gables, Florida 33136 USA; 15 Department of Social Medicine, University of North Carolina School of Medicine, Chapel Hill, NC 27599 USA; 16 Department of Psychology, San Diego State University, San Diego, CA 91910 USA; 17 University of Texas School of Public Health, San Antonio and University of Texas Health Science Center, San Antonio, TX 78229 USA; 18 Section of Cardiovascular Medicine, Boston University Chobanian and Avedisian School of Medicine, Boston, MA 02118 USA; 19 Fred Hutchinson Cancer Research Center, Division of Public Health Sciences, Seattle, WA 98109 USA

**Keywords:** Moderate-to-vigorous physical activity, Physical activity, Hispanics/Latinos, Accelerometry, Longitudinal change

## Abstract

**Background:**

Physical activity promotes health and is particularly important during middle and older age for decreasing morbidity and mortality. We assessed the correlates of changes over time in moderate-to-vigorous physical activity (MVPA) in Hispanic/Latino adults from the Hispanic Community Health Study/Study of Latinos (HCHS/SOL: mean [SD] age 49.2 y [11.5]) and compared them to a cohort of primarily White adults from the Framingham Heart Study (FHS: mean [SD] 46.9 y [9.2]).

**Methods:**

Between 2008 and 2019, we assessed accelerometry-based MVPA at two time points with an average follow-up of: 7.6 y, SD 1.3 for HCHS/SOL, and 7.8 y, SD 0.7 for FHS. We used multinomial logistic regression to relate socio-demographic and health behaviors with changes in compliance with 2018 US recommendations for MVPA from time 1 to time 2 (remained active or inactive; became active or inactive) across the two cohorts.

**Results:**

In HCHS/SOL mean MVPA was 22.6 (SD, 23.8) minutes at time 1 and dropped to 16.7 (19.0) minutes at time 2. In FHS Mean MVPA was 21.7 min (SD, 17.7) at time 1 and dropped to 21.3 min (SD, 19.2) at time 2. Across both cohorts, odds of meeting MVPA guidelines over time were about 6% lower in individuals who had lower quality diets vs. higher, about half in older vs. younger adults, about three times lower in women vs. men, and 9% lower in individuals who had a higher vs. lower BMI at baseline. Cohorts differed in how age, gender, income, education, depressive symptoms, marital status and perception of general health and pain associated with changes in physical activity. High income older Hispanics/Latino adults were more likely to become inactive at the follow-up visit as were HCHS/SOL women who were retired and FHS participants who had lower levels of education and income. Higher depressive symptomology was associated with becoming active only in HCHS/SOL women. Being male and married was associated with becoming inactive in both cohorts. Higher perception of general health and lower perception of pain were associated with remaining active only in FHS adults.

**Conclusions:**

These findings highlight potentially high-risk groups for targeted MVPA intervention.

**Supplementary Information:**

The online version contains supplementary material available at 10.1186/s12889-023-16442-9.

## Background

It is well known that physical activity promotes health and longevity and that it is particularly important during middle and older age for slowing senescence and decreasing morbidity and mortality [1–3]. The Hispanic/Latino population in the US is of great scientific interest because they are the largest minority population in the US and have high levels of risk factors for cardiometabolic disease (such as high rates of diabetes) [4, 5]. As physical activity may be a low cost strategy to mitigate these risk factors [6], this is a unique opportunity to study physical activity longitudinally in this under-studied population.

We conducted a longitudinal study to assess the correlates of physical activity patterns over time in Hispanic/Latino adults from the Hispanic Community Health Study/Study of Latinos (HCHS/SOL) and compared them to a primarily non-Hispanic cohort (comprised primarily of White adults) enrolled in the Framingham Heart Study (FHS). Prior studies in HCHS/SOL and FHS have indicated that lower levels of sedentary behavior as well as higher levels of physical activity and steps are independently associated with more favorable lipids, lower inflammation, and risk of diabetes, particularly in specific sub-cohorts defined by age and glycemic status with effects more robust in older participants and those with greater glucose dysregulation [3, 7–9]. Here we describe socio-cultural and biobehavioral correlates associated with change in moderate-to-vigorous physical activity in the two cohorts. Prior studies indicate age, higher body mass index (BMI), and history of chronic disease, among others as associated with reductions in physical activity [10–13]. We assessed change in moderate-vigorous physical activity (MVPA) and success in meeting the 2018 US aerobic guidelines for MVPA [14] at two assessments approximately eight years apart. Our main goals related to changes in physical activity at two time points as follows:

1. Describe changes in MVPA overall and by sex and age within the two cohorts and
2. Assess the sociocultural and biobehavioral correlates of change in meeting US guidelines for MVPA by sex and age within the two cohorts.

We, therefore, sought to identify individual-level sociodemographic and health-related characteristics in the Hispanic/Latino and primarily non-Hispanic populations that may indicate a propensity for suboptimal physical activity over time to inform targeted health promotion strategies. A strength of this study is integrating a prospective framework of physical activity at two time points.

## Methods

### Study population

**Fig. 1:**
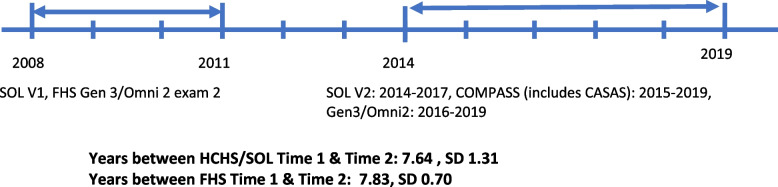
Timeline of physical activity measurements in HCHS/SOL and FHS

In the HCHS/SOL and FHS cohorts, we evaluated physical activity patterns with an average follow-up of 7.64 y, SD 1.31 for HCHS/SOL, and 7.83 y, SD 0.70 for FHS from time 1 to time 2. The first assessment was conducted in 2008–2011 (HCHS/SOL visit 1 and FHS Generation 3 and Omni 2 cohorts, examination 2), and the second assessment in 2017–2019 for HCHS/SOL and 2016–2019 for FHS (See Fig. 1). HCHS/SOL is a prospective population-based study of 16,415 Hispanic/Latino adults aged 18 to 74 years at recruitment who were living in four US metropolitan areas (Bronx, NY; Chicago, IL; Miami, FL; and San Diego, CA) [15, 16]. Recruitment was designed to occur in stable communities to facilitate follow-up and reexaminations. Participants were recruited using a three-stage probability sample design. Following standard protocols, a comprehensive battery of interviews and a clinical assessment with fasting blood draw were conducted by trained and certified staff at in-person clinic visits between 2008 and 2011 (V1). The second visit (V2) period started in October 2014 and concluded in December 2017. In visit 2, 11,623 cohort members were reexamined. Participants underwent accelerometry at V1 examinations, and a subset had these repeated approximately current with V2 (*n* = 4,346). Participants from the Bronx, Chicago, and Miami sites had repeat accelerometry performed beginning in 2017 as part of the Cardiometabolic Outcomes in Multi-ethnic Physical Activity and Sedentary Behavior Study (COMPASS) while repeat accelerometry among participants in the San Diego Field Center was performed beginning in 2015 as part of the CASAS study [17, 18]. Data for daily accelerometer-measured moderate-to-vigorous physical activity (MVPA), sedentary behavior (SB), and light physical activity (LPA) were recorded for up to 7 days using methods described in additional detail below.

**Fig. 2:**
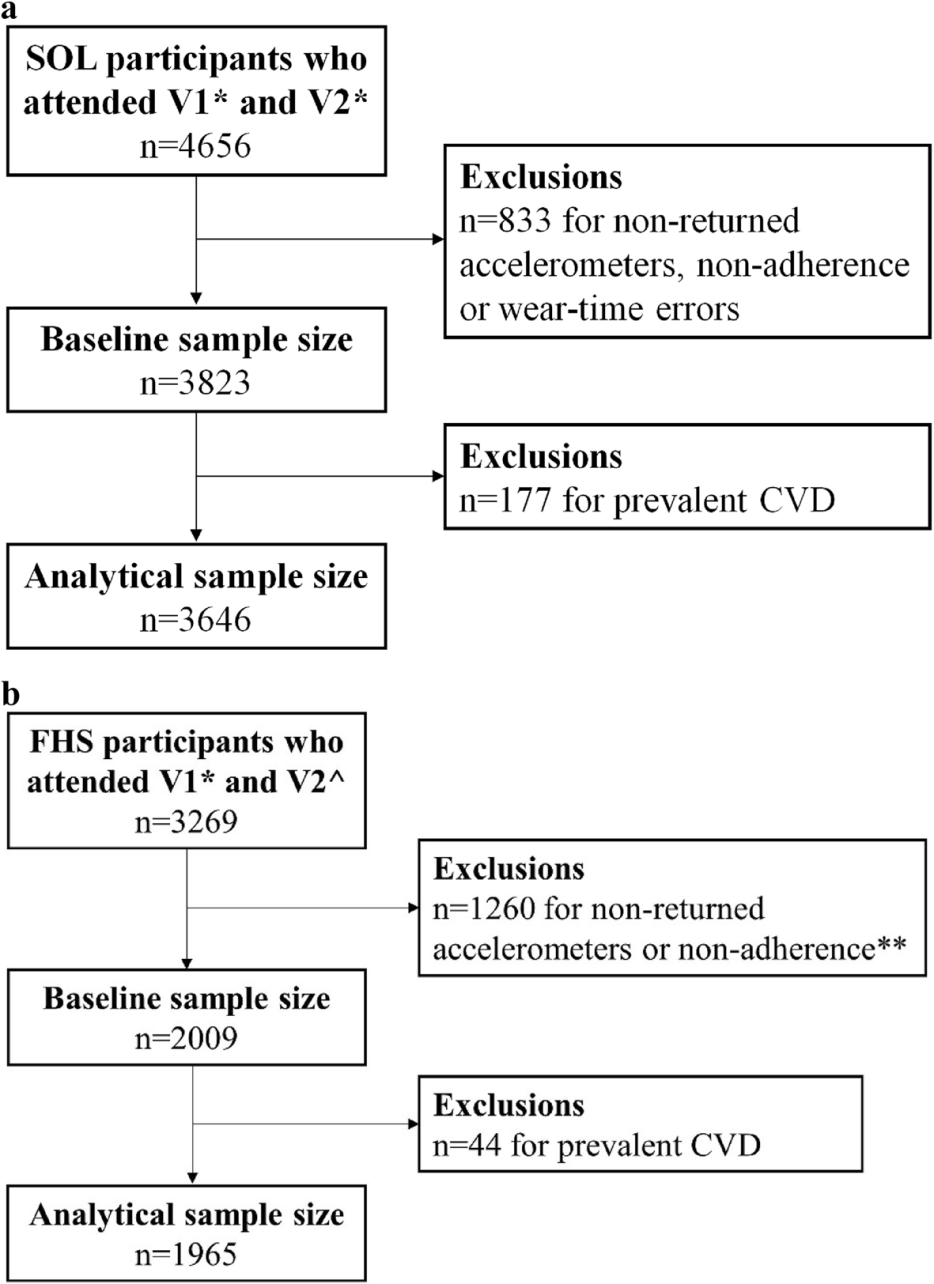
**a** Flow chart for HCHS/SOL. *SOL cohort time 1 (V1) is exam at baseline (2008–2011) and time 2 (V2) is exam at COMPASS and CASAS (2015–2019**)**. **b** Flow chart for FHS. *FHS cohort time 1 (V1) is exam cycle 2 (2008–2011) and ^time 2 (V2) is exam cycle 3 (2016–2019). Cohorts are comprised of Gen, Omni 2 and NOS. **Participants were asked to wear the device for 24 h/day and to only remove it when swimming and bathing. Non-wear time was removed from the data processing and is defined as 60 consecutive minutes of zero counts, allowing for 2-min interruption periods. Adherence is defined as wearing the device for ≥ 10 h/day for at least 3 days, not including the first day of wear, which was excluded from the data processing

Recruitment of FHS Gen 3 (*N* = 4,095), the grandchildren of the 1948 Original Cohort [19, 20] was from 2002 to 2005. In 2002–2005 a minority cohort was also recruited from the Framingham area, the Omni 2 cohort (*N* = 410) and *N* = 103 participants from the NOS (New Offspring Spouses) cohort were also included. Annual contacts were designed to obtain health history updates and maintain contact information. The initial accelerometry measure that we used in this study was obtained during exam cycle 2 (6.5 years after enrollment, 2008–2011) and again during exam cycle 3 (2016–2019). Overall, 3269 FHS participants attended both exam cycles 2 and 3. After excluding those who did not return their accelerometers and/or were non-adherent, 2009 participants (Gen 3 *n* = 1825, Omni 2 *n* = 156, NOS *n* = 28) had complete accelerometry measurements at both exam cycles. See Fig. 2a and b.

### Accelerometry assessment

At both visits and in both cohorts study participants were asked to wear an Actical accelerometer (version B-1; model 198–0200-03 for HCHS/SOL and 198–0200-00 for FHS) [21] on their hip for one week. Details of the accelerometer assessment protocols have been reported previously for FHS [21] and HCHS/SOL [22, 23]. Because we used the same Actical accelerometers during each of the repeat measurement waves, we periodically recalibrated the devices to prevent drift (too many high or low counts). Wear time was identified using the Choi et al. algorithm [24]. Non-wear time was defined as at least 60 consecutive minutes of zero counts, allowing for 2-min interruption periods; nonwear time was removed from data processing. Average counts per minute (cpm) were used to categorize daily minutes in, moderate or vigorous activity by day. Moderate activity was defined as 1535–3961 cpm and vigorous activity as > 3961 cpm [25]. Data from non-adherent participants were excluded, defined as having fewer than 10 h per day and less than three days of accelerometry data. In both cohorts, for those with less than 7 days of wear, we averaged over the adherent days and extrapolated to estimate MVPA for 7 days. In FHS Gen 3/Omni 2/NOS exam 2, participants were asked to wear the device 24 h/day, while participants at other FHS exams and HCHS/SOL were asked to remove the device for sleep. In FHS, we also removed a consecutive 6-h sliding window during each 24-h period which had the lowest total number of accumulated accelerometer counts to remove potential sleep time that was not already removed by the Choi algorithm [24]. In HCHS/SOL, participants were excluded if the accelerometry data were implausible due to technical issues such as daily wear more than 24 h.

### Covariates and potential correlates

Covariates were defined at the time of the initial accelerometry measurement, with a focus on those common to HCHS/SOL and FHS GEN3/Omni2/NOS which included socio-demographics such as age, sex, marital status, education, household income, employment status, and Hispanic/Latino background. Medical history correlates consisted of BMI, glycemic status (prediabetes, diabetes), hypertensive status, and cardiovascular disease (CVD). Health behavior correlates included the Alternative Healthy Eating Index (AHEI-2010) a measure of diet quality that was measured in all HCHS/SOL participants, but only in FHS Gen 3 participants of FHS (i.e. not Omni 2 or NOS). Other variables included smoking, alcohol, and health care use/health insurance. Alcohol use was defined as % of participants consuming alcohol at baseline. We also characterized the use of medications (lipid and glucose-lowering medications, blood pressure medications, and non-steroidal anti-inflammatory drugs). While we did include medications in one of the models, additional adjustments for medication use did not substantially modify the coefficients of other covariates nor were the medications significantly associated with the outcomes. We therefore only present medications as descriptive material in Tables 1 and 2. We also present data related to health insurance as background material.

**Table 1:** Unweighted baseline characteristics overall and according to MVPA guidelines at baseline and follow-up among HCHS/SOL participants

	**Overall**	**Remained active**	**Became active**	**Became inactive**	**Remained inactive**	***p*-value**
**N (%)**	3823 (100.0)	690(18.1)	367 (9.6)	839(22.0)	1927(50.4)	
**Age, mean(SD)**	49.2(11.5)	45.3(11.6)	47.4(10.89)	48.0(11.7)	51.6(11.2)	**< 0.01**
**Sex, %Female**	2501(65.4)	341(49.4)	229(62.4)	487(58)	1444 (74.9)	**< 0.01**
**BMI, mean(SD)**	29.4(5.4)	28.02(4.6)	29.1(5.3)	28.9(5.3)	30.1(5.6)	**< 0.01**
**Change of BMI, mean(SD)**	0.3(2.6)	0.5(2.5)	0.2(2.8)	0.5(2.7)	0.2(2.5)	**< 0.01**
**Center, count (%)**						**< 0.01**
**Bronx**	863(22.6)	257(37.2)	82(22.3)	227(27.1)	297(15.4)	
**Chicago**	867(22.7)	155(22.5)	89(24.3)	206(24.6)	417(21.6)	
**Miami**	684(17.9)	58(8.4)	50(13.6)	122(14.5)	454(23.6)	
**San Diego (CASAS)**	1409(36.9)	220(31.9)	146(39.8)	284(33.8)	759(39.4)	
**Hispanic background, count (%)**						**< 0.01**
**Dominican**	372(9.7)	99(14.4)	43(11.7)	91(10.9)	139(7.2)	
**Central American**	328(8.6)	48(7)	29(7.9)	75(9)	176(9.1)	
**Cuban**	378(9.9)	29(4.2)	22(6)	65(7.8)	262(13.6)	
**Mexican**	1876(49.1)	325(47.2)	195(53.1)	408(48.7)	948(49.2)	
**Puerto Rican**	523(13.7)	126(18.3)	34(9.3)	125(14.9)	238(12.4)	
**South American**	257(6.7)	37(5.4)	31(8.4)	57(6.8)	132(6.9)	
**Other or more than one heritage**	85(2.2)	25(3.6)	13(3.5)	16(1.9)	31(1.6)	
**Years in US, mean(SD)**	23.41(15.3)	22.93(15.0)	22.11(14.6)	22.8(14.9)	24.16(15.8)	**0.01**
**Acculturation, count (%)**						**< 0.01**
**US born**	519(13.6)	141(20.5)	46(12.6)	108(12.9)	224(11.6)	
**Immigration < 10 year**	818(21.4)	133(19.3)	89(24.3)	182(21.7)	414(21.5)	
**Immigration > = 10 year**	2479(65)	414(60.2)	231(63.1)	548(65.4)	1286(66.8)	
**Education, count (%)**						0.48
**Lower than high school**	1446(37.9)	255(37)	127(34.6)	319(38.1)	745(38.7)	
**High school or higher**	2372(62.1)	434(63)	240(65.4)	519(61.9)	1179(61.3)	
**Household Income ($), count (%)**						0.88
**< 20,000 per year**	1621(45.2)	281(43.9)	152(43.1)	362(45.8)	826(45.9)	
**20,000—50,000 per year**	1559(43.5)	282(44.1)	160(45.3)	335(42.4)	782(43.4)	
**> 50,000 year**	404(11.3)	77(12)	41(11.6)	93(11.8)	193(10.7)	
**Marital status, count (%)**						**< 0.01**
**single**	745(19.5)	203(29.5)	63(17.2)	186(22.2)	293(15.2)	
**married**	2223(58.3)	357(51.9)	223(60.8)	470(56.1)	1173(61)	
**widowed or divorced**	848(22.2)	128(18.6)	81(22.1)	182(21.7)	457(23.8)	
**Employment status, count (%)**						**< 0.01**
**retired**	316(8.3)	35(5.1)	23(6.3)	69(8.3)	189(9.9)	
**not employed**	1337(35.2)	207(30.3)	126(34.5)	261(31.4)	743(38.8)	
**part-time work**	729(19.2)	142(20.8)	71(19.5)	158(19)	358(18.7)	
**full-time work**	1413(37.2)	299(43.8)	145(39.7)	343(41.3)	626(32.7)	
**Change of employment status, count (%)**						**< 0.01**
**Stable working hour**	1894(51.1)	368(54.9)	183(52.1)	422(51.7)	921(49.2)	
**Decreasing working hour**	1030(27.8)	148(22.1)	86(24.5)	220(26.9)	576(30.8)	
**Increasing working hour**	785(21.2)	154(23)	82(23.4)	175(21.4)	374(20)	
**AHEI 2010,mean(SD)**	50.85(7.5)	50.95(8.1)	51.22(7.3)	50.89(7.4)	50.6(7.4)	0.35
**Current smoker, count (%)**	610(16.0)	119(17.2)	70(19.2)	136(16.2)	285(14.8)	0.13
**Current alcohol use, count (%)**	1785(46.7)	355(51.4)	169(46)	409(48.7)	852(44.2)	**0.01**
**General Health SF-12**^**a**^**, count (%)**						
**Poor**	124(3.3)	20(2.9)	14(3.8)	20(2.4)	70(3.6)	
**Fair**	907(23.8)	133(19.3)	77(21)	186(22.2)	511(26.6)	
**Good**	1872(49.1)	330(47.9)	186(50.7)	410(49)	946(49.2)	
**Very good**	626(16.4)	147(21.3)	62(16.9)	141(16.9)	276(14.4)	
**Excellent**	284(7.4)	59(8.6)	28(7.6)	79(9.4)	118(6.1)	
** General Health SF-12**^**a**^**, mean (SD)**	2.01(0.9)	2.13(0.9)	2.07(0.9)	2.09(1.0)	1.94(0.9)	**< 0.01**
**General Pain SF-12, count (%)**						**0.03**
**Not at all**	2181(57.2)	421(61.2)	218(59.7)	495(59.1)	1047(54.5)	
**A little bit**	916(24)	155(22.5)	83(22.7)	194(23.2)	484(25.2)	
**Moderately**	368(9.7)	61(8.9)	37(10.1)	85(10.2)	185(9.6)	
**Quite a bit**	252(6.6)	35(5.1)	19(5.2)	41(4.9)	157(8.2)	
**Extremely**	95(2.5)	16(2.6)	8(2.2)	22(2.6)	49(2.5)	
**Health insurance covered, count (%)**	1964(51.8)	387(56.6)	184(50.8)	438(52.6)	955(49.9)	**0.03**
**Health care use in past year, count (%)**	2852(75.4)	501(73.4)	273(74.8)	629(75.6)	1449(76.1)	0.56
**Depressive symptomology, count (%)**	1073(28.3)	197(28.8)	97(26.6)	213(25.6)	566(29.6)	0.15
**Diabetes, count (%)**	454(11.9)	54(7.8)	38(10.4)	79(9.4)	283(14.7)	**< 0.01**
**Hypertension, count (%)**	1150(30.1)	153(22.2)	101(27.5)	226(26.9)	670(34.8)	**< 0.01**
**Medication use, count (%)**						
**AntiDepression medication use**	218(5.8)	39(5.8)	10(2.8)	39(4.7)	130(6.9)	**0.01**
**AntiDiabetes medication use**	171(4.6)	19(2.8)	12(3.3)	28(3.4)	112(5.9)	**< 0.01**
**Lipid Lowering medication use**	420(11.2)	53(7.9)	34(9.4)	79(9.6)	254(13.4)	**< 0.01**
**AntiHypertension medication use**	558(14.9)	64(9.5)	56(15.6)	101(12.2)	337(17.8)	**< 0.01**
**Aspirin use**	844(22.5)	114(17)	71(19.7)	197(23.8)	462(24.4)	**< 0.01**
**Count per minute per day, mean(SD)**						
**Count per minute per day at baseline**	170.6(106.7)	261.8(115)	124.1(50.4)	244.0(113.7)	113.1(47.4)	**< 0.01**
**Count per minute per day at V2**	151.8(98.2)	261.8(112.2)	245.0(101.7)	126.2(51.6)	103.6(49.9)	**< 0.01**
**Change of count per minute per day**	-18.8(115.1)	0.0(142.7)	120.9(106.8)	-117.8(115.6)	-9.5(51.6)	**< 0.01**
**Sedentary time (minute/day), mean(SD)**						
**Sedentary time at baseline**	698.3(99.7)	652.9(100.0)	723.7(88.5)	655.5(100.3)	728.4(88.6)	**< 0.01**
**Sedentary time at V2**	711.7(101.8)	649.4(103.5)	657.2(100.5)	719.0(92.9)	744.0(89.1)	**< 0.01**
**Change of sedentary time**	13.4(107.5)	-3.5(115.3)	-66.5(113.7)	63.5(109.4)	15.6(89.1)	**< 0.01**
Light activity* (minute/day), mean(SD)						
**Light activity at baseline**	238.9(90.1)	261.3(91.3)	224.9(86.5)	263.5(92.0)	222.9(86.0)	**< 0.01**
**Light activity at V2**	229.4(92.5)	264.1(95.9)	259.7(94.9)	229.3(89.4)	208.8(86.3)	**< 0.01**
**Change of light activity**	-9.5(96.6)	2.7(101.6)	34.8(108.9)	-34.2(99.6)	-14.1(85.8)	**< 0.01**
**MVPA (minute/day), mean(SD)**						
**MVPA at baseline**	22.6(23.8)	46.7(25.8)	11.0 (5.8)	40.5(23.7)	8.0(5.8)	**< 0.01**
**MVPA at V2**	16.7(19.0)	41.7(19.8)	38.3(19.5)	10.0(6.4)	6.3(5.7)	**< 0.01**
**Change of MVPA**	-5.9(24.4)	-4.9(29.6)	27.3(20.4)	-30.5(24.1)	-1.8(6.9)	**< 0.01**

^a^General health SF-12 is treated on a continuous scale: 1 = Poor, 2 = Fair, 3 = Good, 4 = Very good, 5 = Excellent

^*^Standardized light physical activity to 16-h day using residuals

**Table 2:** Unweighted baseline characteristics overall and according to MVPA guidelines at baseline and follow-up among FHS participants

	**Overall**	**Remained active**	**Became active**	**Became inactive**	**Remained inactive**	***p*-value**
**N (%)**	2009	572 (28.5)	280 (13.9)	330 (16.4)	827 (41.2)	
**Age, mean(SD)**	46.9 (9.2)	44.9 (8.8)	45.6 (8.3)	46.3 (8.4)	49 (9.6)	**< 0.01**
**Sex, % Female**	1066 (53.1)	302 (52.8)	150 (53.6)	155 (47)	459 (55.5)	0.07
**BMI, mean(SD)**	27.5 (5.2)	25.7 (4.2)	26.9 (4.8)	27.3 (4.7)	29 (5.7)	**< 0.01**
**Change in BMI, mean(SD)**	0.6 (2.4)	0.5 (2.1)	0.4 (2.0)	1.1 (2.2)	0.6 (2.6)	**< 0.01**
**Hispanic background, count (%)**	65 (3.3)	10 (1.8)	5 (1.8)	12 (3.7)	38 (4.7)	**0.01**
**Education, count (%)**						**< 0.01**
**Lower than high school (or GED)**	250 (12.5)	27 (4.7)	28 (10)	52 (15.8)	143 (17.3)	
**High school or higher**	1758 (87.5)	545 (95.3)	252 (90)	278 (84.2)	683 (82.7)	
**Household Income ($), count (%)**						**< 0. 01^**
**< 20,000 per year**	71 (3.5)	15 (2.6)	5 (1.8)	12 (3.6)	39 (4.7)	
**20,000—50,000 per year**	364 (18.1)	71 (12.4)	39 (13.9)	66 (20)	188 (22.7)	
**> 50,000 year**	1503 (74.8)	460 (80.4)	231 (82.5)	244 (73.9)	568 (68.7)	
**Unknown**	71 (3.5)	26 (4.6)	5 (1.8)	8 (2.4)	32 (3.9)	
**Marital status, count (%)**						**< 0.01**
**single**	266 (13.3)	102 (17.8)	25 (8.9)	40 (12.2)	99 (12)	
**married**	1480 (73.8)	423 (74)	225 (80.4)	247 (75.1)	585 (70.9)	
**widowed or divorced**	260 (13)	47 (8.2)	30 (10.7)	42 (12.8)	141 (17.1)	
**Employment status, count (%)**						**< 0.01**
**retired**	58 (2.9)	12 (2.1)	3 (1.1)	6 (1.8)	37 (4.5)	
**not employed**	194 (9.7)	41 (7.2)	33 (11.8)	27 (8.2)	93 (11.3)	
**part-time work**	278 (13.9)	83 (14.5)	43 (15.4)	39 (11.9)	113 (13.7)	
**full-time work**	1474 (73.6)	436 (76.2)	200 (71.7)	257 (78.1)	581 (70.5)	
**Change of employment status, count (%)**						0.07
** Stable working hour**	1380 (72)	429 (75.4)	192 (69.3)	243 (74.5)	563 (69.1)	
**Decreasing working hour**	317 (16.5)	82 (14.4)	44 (15.9)	48 (14.7)	156 (19.1)	
**Increasing working hour**	219 (11.4)	58 (10.2)	41 (14.8)	35 (10.7)	96 (11.8)	
**AHEI 2010,mean(SD)**	61.4 (13.1)	64.2 (12.5)	61.3 (13.3)	61.2 (13.7)	59.3 (12.9)	**< 0.01**
**Current smoker, count (%)**	137 (6.8)	19 (3.3)	17 (6.1)	21 (6.4)	80 (9.7)	**< 0.01**
**Current alcohol use, count (%)**	1651 (83.8)	495 (88.6)	239 (86.6)	266 (83.1)	651 (80%)	**< 0.01**
**General Health SF-12**^**a**^**, count (%)**						**< 0.01**
**Poor**	6 (0.3)	0 (0)	0 (0)	0 (0)	6 (0.7)	
**Fair**	41 (2)	5 (0.9)	5 (1.8)	8 (2.4)	23 (2.8)	
**Good**	455 (22.7)	79 (13.8)	57 (20.4)	67 (20.3)	252 (30.5)	
**Very good**	961 (47.9)	252 (44.1)	145 (51.8)	162 (49.1)	402 (48.7)	
**Excellent**	545 (27.1)	236 (41.3)	73 (26.1)	93 (28.2)	143 (17.3)	
**General Health SF-12**^**a**^**,** **mean (SD)**	3.0 (0.8)	3.3 (0.7)	3.0 (0.7)	3.0 (0.8)	2.8 (0.8)	**< 0.01**
**General Pain SF-12, count (%)**						**< 0.01**
**Not at all**	1310 (65.2)	424 (74.1)	193 (68.9)	213 (64.6)	480 (58.1)	
**A little bit**	515 (25.7)	122 (21.3)	63 (22.5)	88 (26.7)	242 (29.3)	
**Moderately**	121 (6)	20 (3.5)	16 (5.7)	19 (5.8)	66 (8)	
**Quite a bit**	52 (2.6)	6 (1.1)	7 (2.5)	9 (2.7)	30 (3.6)	
**Extremely**	10 (0.5)	0 (0)	1 (0.4)	1 (0.3)	8 (1)	
**Health insurance covered, count (%)**	1994 (99.3)	572 (100)	279 (99.6)	327 (99.1)	816 (98.7)	**0.01**
**Health care use in past year, count (%)**	1890 (94.1)	536 (93.7)	262 (93.6)	314 (95.2)	778 (94.1)	0.81
**Depressive symptomology, count (%)**	127 (6.3)	27 (4.7)	19 (6.8)	20 (6.1)	61 (7.4)	0.24
**Diabetes, count (%)**	88 (4.4)	10 (1.8)	6 (2.2)	10 (3)	62 (7.5)	**< 0.01**
**Hypertension, count (%)**	422 (21)	83 (14.5)	39 (13.9)	60 (18.2)	240 (29)	**< 0.01**
**Medication use, count (%)**						
**Anti-diabetes medication use**	56 (2.8)	4 (0.7)	4 (1.4)	7 (2.1)	41 (5)	**< 0.01**
**Lipid Lowering medication use**	316 (15.7)	61 (10.7)	32 (11.4)	58 (17.6)	165 (20)	**< 0.01**
**AntiHypertension medication use**	322 (16.1)	58 (10.2)	29 (10.4)	46 (14.1)	189 (23)	**< 0.01**
**Aspirin use**	302 (15.1)	56 (9.8)	37 (13.3)	56 (17)	153 (18.5)	**< 0.01**
**Count per minute per day,mean(SD)**						
**Count per minute per day at baseline**	170.0 (88.8)	249.6 (88.8)	124.9 (38.1)	217.4 (76.5)	111.1 (38.2)	**< 0.01**
**Count per minute per day at V2**	176.7 (97.9)	267.3 (93.0)	235.1 (94.1)	132.3 (43.6)	112.0 (42.0)	**< 0.01**
**Change of count per minute per day**	6.8 (95.1)	17.7 (105.5)	110.1 (96.2)	-85.1 (77.9)	0.9 (40.8)	**< 0.01**
**Sedentary time (mean minute/day),mean(SD)**						
**Sedentary time at baseline**	727.4 (68.5)	708.7 (65.5)	738.7 (64.6)	701.5 (74.9)	746.8 (62.3)	**< 0.01**
**Sedentary time at V2**	708.4 (65.6)	684.9 (60.2)	692.5 (69.7)	715.6 (61.7)	727.1 (62.9)	**< 0.01**
**Change of sedentary time**	-19.0 (67.4)	-23.8 (64.9)	-46.2 (66.2)	14.2 (74.7)	-19.7 (61.0)	**< 0.01**
Light activity* (mean minute/day),mean(SD)						
**Light activity at baseline**	211.6 (63.4)	213.4 (62.0)	210.2 (63.0)	227.3 (69.7)	204.4 (60.6)	**< 0.01**
**Light activity at V2**	224.2 (60.6)	229.5 (58.2)	226.7 (63.4)	227.5 (60.0)	218.5 (61.1)	**< 0.01**
**Change of light activity**	12.7 (61.0)	16.1 (59.6)	16.5 (58.3)	0.2 (69.3)	14.0 (58.7)	**< 0.01**
**MVPA(mean minute/day),mean(SD)**						
**MVPA at baseline**	21.7 (17.7)	38.7 (16.5)	11.5 (5.0)	32.8 (14.6)	8.9 (5.3)	** 0.01**
**MVPA at V2**	21.3 (19.2)	40.7 (17.6)	34.1 (16.5)	11.1 (5.5)	7.6 (5.6)	**< 0. 01**
**Change of MVPA**	-0.3 (19.0)	2.0 (20.3)	22.5 (17.1)	-21.7 (15.2)	-1.2 (6.5)	**< 0. 01**

^a^General health SF-12 is treated on a continuous scale: 1 = Poor, 2 = Fair, 3 = Good, 4 = Very good, 5 = Excellent

^Excludes ‘Unknown’ household income

^*^Standardized light physical activity to 16-h day using residuals

In both HCHS/SOL and FHS cohorts, variables such as BMI were repeatedly assessed at scheduled visits however, for some variables such as SF-12 General Health, it was evaluated only at V1 for HCHS/SOL. In the fully adjusted Model 3 for both cohorts, change in BMI and change in work hours were the only variables that were assessed for change using baseline and follow-up visits. All other variables included in the model were ascertained at baseline. For analyses related to a change in work hours in HCHS/SOL, we used the following definitions using variables from V1 & V2:

- *Decreasing work hours*: ^1^Full-time → Part-time/Not employed/Retired; ^2^Part-time → Not employed/Retired; ^3^Not employed → Retired.
- *Stable work hours*: No change in status as Retired; Not employed; Part-time work; Full-time work.
- *Increasing work hours*: ^1^Part-time/Not employed/Retired → Full-time; ^2^Not employed/Retired → Part-time; ^3^Retired → Not employed. The reason for including not employed in the “increasing work hour category” is that we assumed this group of individuals was looking for work and more likely to be active than the retired group.

The variable prediabetes was defined as: Fasting blood glucose (FBG) levels 100 mg/dL ≤ FBG < 126 mg/dL and Hemoglobin A1c (HbA1c) ≤ 6.5 -or- 5.7 ≤ HbA1c ≤ 6.5 and FBG < 126. If HbA1c > 6.5 then participants were categorized as diabetic. Depressive symptomology was defined as CES-D20—score ≥ 16 in FHS and CES-D10 ≥ 10 as depressive symptomology for HCHS/SOL [26]. Scores ≥ 10 on the CES-D10 or ≥ 16 on the CES-D20 denote elevated depressive symptoms which may reflect clinical depression but are not equivalent to a clinical diagnosis [27, 28].

### Statistical analyses

Participants were defined as meeting the 2018 US Physical Activity Guidelines [14] at baseline (V1) and follow-up (V2) if they had ≥ 150 min of moderate-intensity activity a week, ≥ 75 min of vigorous-intensity physical activity a week, or the equivalent combination of moderate and vigorous activity (1). To evaluate change in physical activity, we focused on whether 2018 US guidelines for MVPA were met at the first and second assessments in HCHS/SOL and FHS by using multinomial logistic regression with four possible longitudinal outcomes: (1) remained inactive or did not meet 2018 guidelines at both visits, (2) became active at V2 (changed from not meeting MVPA guidelines at V1 to meeting MVPA guidelines at V2), (3) became inactive at V2 (changed from meeting MVPA guidelines at V1 to not meeting guidelines at V2) and (4) remained active or consistently met guidelines at both visits. We used multinomial logistic regression to model this 4-level change in physical activity in relation to sociodemographic and medical history covariates in HCHS/SOL and FHS separately. Our primary interest lies in three contrasts: remained active vs. remained inactive: the comparison between these groups of individuals reflects the largest persistent difference in MVPA levels over time (OR > 1 indicates more likely persistently active than persistently inactive); became active vs. remained inactive: these individuals’ level of MVPA improves over time (OR > 1 indicates more likely to change from inactive to active), and lastly became inactive vs. remained active: these individuals’ level of MVPA worsens over time (OR > 1 indicates more likely to change from active to inactive).

We fitted the following nested models. Model 0 (M0) includes age and sex. Model 1 (M1) includes covariates in M0 plus BMI at V1, change in BMI from V1 to V2, Hispanic/Latino background (HCHS/SOL cohort), household income, and education. Model 2 (M2) includes covariates in M1 plus marital status, employment status, change in work hours, AHEI- 2010, smoking, and alcohol consumption. Model 3 (M3; primary model) includes the addition of the following medical history variables: SF-12 General Health, SF-12 Pain, depression status, diabetes, and hypertensive status at baseline. Models were stratified by sex and age (< 50, ≥ 50 years old). Education level was categorized as equal to or greater than high school (HS; or GED) compared to less than HS. Household income was treated on a continuous scale: 1 = $20,000, 2 = $20,000-$50,000 3 ≥ $50,000 (i.e., each unit increase signifies higher income) as was a change in work hours: 1 = decreasing, 2 = stable, 3 = increasing. Self-reported health was coded as 0 = Poor, 1 = Fair, 2 = Good, 3 = Very good, and 4 = Excellent.

To fully examine other factors associated with MVPA levels over time in addition to age and sex, we also conducted age and sex-stratified analyses. Each cohort was analyzed separately. To take into consideration multiple contrasts, we conservatively considered a 2-sided value of *P* < 0.01 as statistically significant for all models. We also discuss results that had moderate or suggestive evidence of association defined by the 0.01 < *P* < 0.10 level. All analyses were performed using SAS 9.4 (Cary NC) and R version 4.0.5. Since HCHS/SOL has a probability sample design all HCHS/SOL models were additionally weighted to the 2010 US Census population. FHS models were unweighted given that FHS did not use a population-based sampling frame.

## Results

### Study sample

**Fig. 3:**
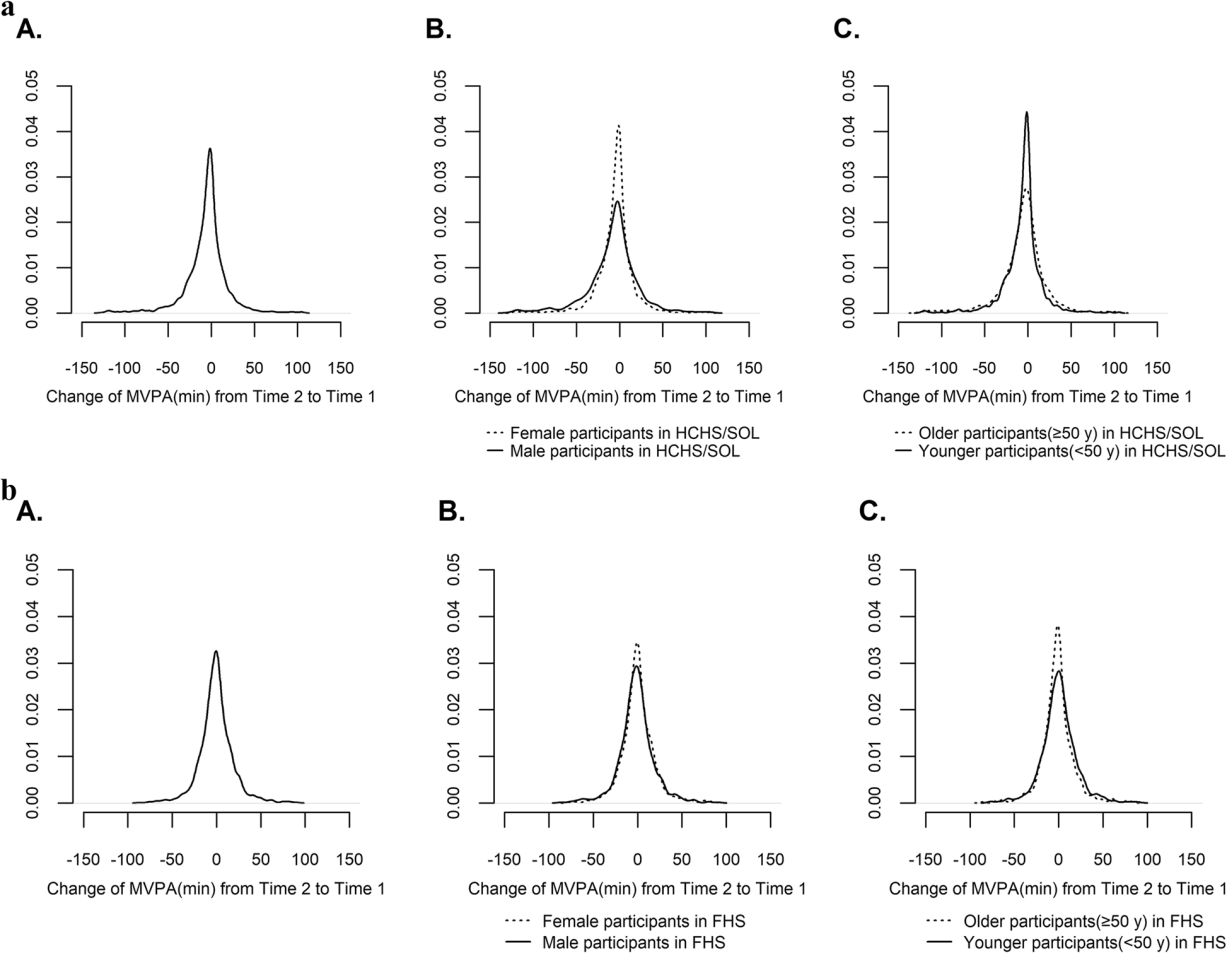
**a** Change in MVPA from Time 2 to Time 1 in HCHS/SOL. **b** Change in MVPA from Time 2 to Time 1 in FHS

There were 3823 participants in the HCHS/SOL cohort and 2009 participants in the FHS cohort (Fig. 2) who were adherent at both assessments and had plausible accelerometry measures (Fig. 2). Excluding HCHS/SOL participants with CVD and two participants with unknown/missing CVD status at baseline (total *n* = 177), the final sample size is *n* = 3646. Excluding FHS participants with CVD at baseline (*n* = 44) the final sample size is *n* = 1965. Figures 3a & b show change in MVPA from time 1 to time 2 in HCHS/SOL and FHS cohorts.

Socio-demographic and medical history characteristics for HCHS/SOL and FHS cohort, overall and stratified by the four physical activity outcome categories, are reported in Tables 1 and 2. Overall participants in HCHS/SOL were older than FHS participants ((unweighted mean age of 49.2 yrs (SD, 11.5) with age range of 18 to 74 yr vs. 46.9 yrs (SD, 9.2) with age range of 24 to 83 yr and had slightly higher mean BMI. The proportion of women was similar between the two cohorts. Overall fewer HCHS/SOL than FHS participants had equal or greater than a high school education (62.1% vs. 87.5%, respectively). Additionally fewer HCHS/SOL than FHS participants were married (58.3% vs. 73.8%, respectively). As for employment, 37.2% of HCHS/SOL participants were employed full-time compared with 73.6% of FHS participants. Only 11.3% of HCHS/SOL participants had an annual income of more than $50,000 compared with 74.8% of FHS participants. The number of participants with pre-diabetes was higher in HCHS/SOL than in FHS (46.8% vs. 21.5%, respectively), and more HCHS/SOL than FHS participants had hypertension (30.1% vs. 21%, respectively). The mean diet quality score based on AHEI-2010 was lower in HCHS/SOL compared to FHS participants (data only available from Gen3 participants in FHS) ((50.9 (SD,7.5) vs. 61.4 (SD, 13.1)); more HCHS/SOL than FHS participants were current smokers (16% vs. 6.8%) and fewer HCHS/SOL than FHS participants drank alcohol (46.7% vs 83.8%, respectively). The perception of overall health in HCHS/SOL participants was lower compared to FHS (mean SF12-General Health of 2.0 (0.9) compared to 3.0 (0.8) in FHS) and more FHS than HCHS/SOL participants mentioned having no pain at all (65.2% vs 57.2%). About 6.3% of FHS participants compared to 28.3% of HCHS/SOL participants described having a higher burden of depressive symptoms.

Neither average accelerometer counts nor minutes of MVPA differed between HCHS/SOL Hispanics/Latinos and FHS non-Hispanics/Latinos (Tables 1 and 2). A higher proportion of FHS than HCHS/SOL participants were persistently active or became active over time, and conversely more HCHS/SOL than FHS participants became inactive or remained inactive, after adjusting for sex and age (Additional Table 1). The proportions who fell into activity groups were similar. For HCHS/SOL participants without CVD at baseline (analytical sample) or *n* = 3646, 1474/3646 = 40.43% met the MVPA guidelines at baseline and 1024/3646 = 28.09% met the MVPA guidelines at follow-up. For FHS excluding participants with CVD at baseline (analytical sample), *N* = 1965, 887/1965 = 45.14% met the MVPA guidelines at baseline and 840/1965 = 42.75% met the MVPA guidelines at follow-up.

### Association of age and sex with change in physical activity from V1 to V2 in HCHS/SOL and FHS

Tables 3 and 4 first show minimally-adjusted (age and sex only) multinomial logit models. These results indicate the importance of younger age and being male in HCHS/SOL in remaining persistently active compared to remaining inactive at both time points. Specifically, older age (10 y increase in age) is associated with almost 40% higher odds of staying inactive vs. active over time and men had three times the odds of staying active over time compared with women. Somewhat similar trends are seen in FHS for age, but not for men. In reviewing the multivariable-adjusted results, we focus our discussion on the multivariable-adjusted Model 3 in Tables 3 and 4. Additional Table 2a & b show Models 0 to 2.

**Table 3:** Association of socio-demographic and biobehavioral variables with change in MVPA over time in HCHS/SOL

Variables	Remained Active vs Remained Inactive		Became Active vs Remained Inactive		Became Inactive vs Remained Active	
	OR (95% CI)	*p*-value	OR (95% CI)	*p*-value	OR (95% CI)	*p*-value
**Minimally adjusted model (M0)**						
Age (10 yr. units)	0.57(0.51,0.65)	**< 0.01**	0.71(0.63,0.81)	**< 0.01**	1.37(1.21,1.57)	**< 0.01**
Sex [Men vs Women]	3.44(2.40,4.94)	**< 0.01**	1.77(1.13,2.76)	**0.01**	0.63(0.43,0.91)	**0.01**
**Fully Adjusted model (M3)**						
Age (10 yr. increments)	0.54(0.44,0.66)	**< 0.01**	0.76(0.57,1)	**0.05**	1.29(1.04,1.61)	**0.02**
Sex [Men vs Women]	3.52(2.23,5.54)	**< 0.01**	1.76(1.05,2.95)	**0.03**	0.59(0.36,0.96)	**0.03**
BMI at baseline (1 kg/m^2^ increments)	0.95(0.91,0.99)	**0.01**	1(0.95,1.06)	0.96	1.02(0.99,1.06)	0.18
Change in BMI (1 kg/m^2^ increments)	0.97(0.91,1.02)	0.23	1(0.9,1.11)	0.93	1.07(0.99,1.16)	0.11
Household income^a^	0.95(0.75,1.21)	0.67	0.9(0.65,1.27)	0.56	0.99(0.73,1.33)	0.92
Education [Greater than HS vs HS (or GED)/Less than HS]	0.81(0.6,1.1)	0.18	0.93(0.56,1.55)	0.79	1.09(0.73,1.64)	0.67
Marital Status		**0.02**		0.37		0.37
Married/Living with partner vs Single	0.6(0.41,0.89)	**0.01**	1.26(0.68,2.3)	0.46	1.4(0.88,2.22)	0.15
Separated/Widowed/Divorced vs Single	0.96(0.52,1.76)	0.89	1.45(0.61,3.45)	0.40	1.15(0.66,1.99)	0.62
Employment status at baseline		0.29		0.22		0.11
Full time vs Retired	1.54(0.56,4.26)	0.40	2.27(0.97,5.33)	0.06	0.53(0.18,1.56)	0.25
Part time vs Retired	1.9(0.74,4.92)	0.19	1.61(0.74,3.5)	0.23	0.37(0.14,0.95)	**0.04**
Unemployed vs Retired	1.35(0.57,3.21)	0.49	1.94(0.89,4.23)	0.10	0.43(0.17,1.04)	0.06
Change in work hours^b^	1.11(0.8,1.54)	0.53	1.53(0.96,2.41)	0.07	0.95(0.64,1.4)	0.78
AHEI 2010	1.08(1.04,1.12)	**< 0.01**	1.03(0.97,1.08)	0.37	0.97(0.93,1)	**0.05**
Current smoking status	1.27(0.77,2.1)	0.34	1.73(0.95,3.17)	0.07	0.69(0.41,1.17)	0.17
Current alcohol consumption	1.04(0.72,1.5)	0.83	1.06(0.69,1.62)	0.80	1.09(0.71,1.66)	0.70
General Health SF-12	1.18(0.97,1.43)	0.10	1.09(0.82,1.44)	0.56	0.94(0.73,1.22)	0.65
General Pain SF-12	0.98(0.83,1.17)	0.83	0.95(0.76,1.18)	0.62	1.02(0.84,1.23)	0.88
Depressive symptomology	1.19(0.86,1.65)	0.29	1.13(0.67,1.91)	0.66	0.65(0.39,1.08)	0.09
Diabetes status	1.08(0.57,2.06)	0.81	0.83(0.39,1.75)	0.62	0.55(0.28,1.09)	0.08
Hypertensive status	1.2(0.77,1.88)	0.42	0.99(0.6,1.63)	0.96	1.02(0.63,1.65)	0.94

^a^Household income is treated on a continuous scale: 1 =  < $20000, 2 = $20000–50000, 3 =  ≥ $50,000 (i.e., each unit increase signifies higher income)

^b^Change in work hours is treated on a continuous scale: -1 = Decreasing, 0 = Stable, 1 = Increasing

**Table 4:** Association of socio-demographic and biobehavioral variables with change in MVPA over time in in FHS

Variables	Remained Active vs Remained Inactive		Became Active vs Remained Inactive		Became Inactive vs Remained Active	
	OR (95% CI)	*p*-value	OR (95% CI)	*p*-value	OR (95% CI)	*p*-value
**Minimally adjusted model (M0)**						
Age (10 yr. units)	0.61 (0.54, 0.69)	**< 0.01**	0.68 (0.58, 0.79)	**< 0.01**	1.20 (1.03, 1.40)	**0.02**
Sex [Men vs Women]	1.11 (0.89, 1.38)	0.36	1.10 (0.83, 1.45)	0.50	1.24 (0.94, 1.63)	0.13
**Fully Adjusted model (M3)**						
Age (10 yr. increments)	0.63 (0.53, 0.76)	**< 0.01**	0.70 (0.57, 0.87)	**< 0.01**	1.21 (0.98, 1.51)	0.08
Sex [Men vs Women]	2.11 (1.55, 2.87)	**< 0.01**	1.62 (1.14, 2.30)	**0.01**	0.93 (0.66, 1.33)	0.70
BMI at baseline (1 kg/m^2^ increments)	0.88 (0.86, 0.91)	**< 0.01**	0.94 (0.91, 0.98)	**< 0.01**	1.08 (1.04, 1.13)	**< 0.01**
Change in BMI (1 kg/m^2^ increments)	0.92 (0.87, 0.98)	**0.01**	0.90 (0.84, 0.96)	**< 0.01**	1.15 (1.07, 1.24)	**< 0.01**
Household income^a^	1.27 (0.92, 1.76)	0.14	1.41 (0.96, 2.06)	0.08	0.71 (0.49, 1.02)	0.07
Education [Greater than HS vs HS (or GED)/Less than HS]	2.66 (1.58, 4.47)	**< 0.01**	1.34 (0.82, 2.19)	0.25	0.34 (0.19, 0.59)	**< 0.01**
Marital Status		**< 0.01**		0.15		0.09
Married/Living with partner vs Single	0.74 (0.49, 1.13)	0.16	1.59 (0.90, 2.81)	0.11	1.69 (1.02, 2.81)	**0.04**
Separated/Widowed/Divorced vs Single	0.42 (0.24, 0.73)	**< 0.01**	1.13 (0.57, 2.24)	0.73	1.88 (0.97, 3.66)	0.06
Employment status at baseline		0.50		0.63		0.93
Full time vs Retired	1.25 (0.41, 3.76)	0.70	1.63 (0.34, 7.77)	0.54	1.17 (0.27, 5.01)	0.84
Part time vs Retired	1.25 (0.40, 3.89)	0.70	2.12 (0.43, 10.36)	0.36	0.98 (0.22, 4.37)	0.98
Unemployed vs Retired	0.82 (0.25, 2.69)	0.74	1.68 (0.33, 8.48)	0.53	1.09 (0.23, 5.17)	0.92
Change in work hours^b^	0.96 (0.71, 1.30)	0.80	1.00 (0.70, 1.41)	0.99	1.14 (0.79, 1.65)	0.50
AHEI 2010^c^	1.04 (1.02, 1.05)	**< 0.01**	1.02 (1.01, 1.03)	**0.01**	0.98 (0.97, 1.00)	**0.01**
Current smoking status	0.46 (0.25, 0.83)	**0.01**	0.68 (0.36, 1.26)	0.22	1.25 (0.61, 2.58)	0.54
Current alcohol consumption	1.19 (0.79, 1.77)	0.40	1.16 (0.74, 1.83)	0.52	0.83 (0.52, 1.33)	0.44
General Health SF-12	1.43 (1.17, 1.74)	**< 0.01**	1.14 (0.91, 1.43)	0.27	0.90 (0.71, 1.14)	0.38
General Pain SF-12	0.70 (0.57, 0.86)	**< 0.01**	0.88 (0.72, 1.09)	0.25	1.34 (1.06, 1.70)	**0.01**
Depressive symptomology	1.54 (0.82, 2.88)	0.18	1.86 (0.98, 3.51)	0.06	0.82 (0.39, 1.71)	0.59
Diabetes status	0.61 (0.25, 1.52)	0.29	0.61 (0.22, 1.67)	0.33	1.39 (0.47, 4.11)	0.55
Hypertensive status	0.94 (0.64, 1.38)	0.76	0.57 (0.36, 0.91)	**0.02**	0.85 (0.54, 1.35)	0.49

Inclusion of the variable for Hispanic/Latino background led to unstable estimates and is subsequently excluded from this model

^a^Household income is treated on a continuous scale: 1 =  < $20000, 2 = $20000–54999, 3 =  ≥ $55,000 (i.e., each unit increase signifies higher income)

^b^Change in work hours is treated on a continuous scale: 1 = Decreasing, 2 = Stable, 3 = Increasing

^c^AHEI 2010 is measured only for Gen 3 participants (*N* = 1629)

#### HCHS/SOL

Among Hispanics/Latinos (Tables 3), first, we examined the contrast between the remained active group versus the remained inactive group. Using the *P* < 0.01 criterion, four variables were associated with consistently having a level of MVPA that met guidelines, vs. consistently not meeting MVPA guidelines: male sex, OR = 3.52 (95% CI: 2.23, 5.54) vs. female, younger age OR = 0.54 (95% CI: 0.44,0.66) per 10 yr increment, lower BMI, OR = 0.95 (95% CI: 0.91,0.99) per 1 kg/m^2^ increment, and higher AHEI-2010 score, OR = 1.08 (95% CI: 1.04, 1.12) per unit increment. The importance of age and sex is also suggested by other analyses in HCHS/SOL. The odds of becoming active vs. remaining inactive were higher for HCHS/SOL men than for HCHS/SOL women OR = 1.77 (95% CI: 1.13, 2.76) and conversely the odds of becoming inactive vs. remaining active were lower for HCHS/SOL men than for HCHS/SOL women, OR = 0.63 (95% CI: 0.43, 0.91). Older individuals were more likely to become inactive between visits ((OR = 1.29 (95% CI: 1.04, 1.61)).

#### FHS

According to Table 4 odds of remained active vs. remained inactive with respect to the sex, age, BMI and AHEI-2010 were as follows: being male, OR = 2.11 (95% CI: 1.55, 2.87) vs. females, younger age, OR = 0.63 (95% CI: 0.53, 0.76) per 10-year increment, lower BMI, OR = 0.88 (95% CI: 0.86, 0.91) per 1 kg/m^2^ increment from baseline, and higher AHEI-2010 score, OR = 1.04 (95% CI: 1.02, 1.05) per unit increase. In addition, greater than high school education, OR = 2.66 (95% CI: 1.58, 4.47) vs. less than high school education, and being separated/widowed/divorced, OR = 0.42 (95% CI: 0.24,0.73), vs. being single and higher perception of General Health SF-12, OR = 1.43 (95% CI: 1.17, 1.74) per unit increase were also associated with odds of remaining active per MVPA guidelines. In contrast to HCHS/SOL, among participants in FHS, higher SF-12 pain was associated with significantly lower odds of remaining active, OR = 0.70 (95% CI: 0.57,0.86).

### Sex-specific analyses

#### HCHS/SOL

The association of age, baseline BMI, and diet with remaining active did not appear to differ considerably between the two sexes (Additional Table 3a). However, a greater increase in BMI over time was associated with significantly lower odds of remaining active over time OR = 0.92 (95% CI: 0.86, 0.99) in women, but not men OR = 1.04 (95% CI: 0.93, 1.17). This outcome also had a strong and consistent association with a more favorable diet in both men and women (per unit of AHEI, OR = 1.09 (95% CI: 1.03,1.15) in males and OR = 1.08 (95% CI: 1.03, 1.14) in females.

Additionally baseline BMI had a suggestive association with the remaining active vs. remaining inactive contrast in both sexes, OR per unit BMI = 0.94 (95% CI: 0.88–1) in men and 0.95 (95% CI: 0.90, 0.99) in women.

Among Hispanic/Latino men, but not Hispanic/Latino women being married/living with a partner as compared with being single was associated with lower odds of remaining persistently active, OR = 0.34 (95% CI: 0.17, 0.67). Among Hispanic/Latino women, but not men, the odds of remaining active were especially high for part-time workers, OR = 4.17 (95% CI: 1.12, 15.63) vs. retired), *P* < 0.05) and in participants with depressive symptomology, OR = 1.89 (95% CI: 1.13, 3.15), *P* < 0.05. Conversely, the odds of becoming inactive were lower for women with part-time work, OR = 0.22 (95% CI: 0.06, 0.83) vs. retired, *P* < 0.05 and having depressive symptomology, OR = 0.39 (95% CI: 0.2, 0.73), *P* < 0.01. Additionally the odds of becoming inactive were lower for women who were unemployed vs. retired, OR = 0.25 (95% CI: 0.08,0.81), *P* < 0.05.Among Hispanic/Latino women, while not statistically significant, the results suggested the presence of an association between higher SF-12 pain and lower odds of remaining active, OR = 0.81 (95% CI: 0.66, 1.01), *P* = 0.06; this association was not apparent in Hispanic/Latino men.

#### FHS

For both men and women in FHS as in HCHS/SOL, age, BMI, and AHEI-2010 were strongly associated with remaining persistently active (See Additional Table 3b). Additionally education in men OR = 3.29 (95% CI: 1.58, 6.83) and in women OR = 2.18 (95% CI: 1.03, 4.61), and general perception of overall health, in men OR = 1.50 (95% CI: 1.12, 2.00) and in women OR = 1.32 (95% CI: 0.99, 1.74) were also associated with remaining persistently active for both sexes. There were also suggestive differences by sex with smoking with men, but not women, having lower odds of remaining persistently active, OR = 0.44 (95% CI: 0.19,0.99), *P* = 0.05; as well being separated/widowed/divorced vs. single particularly among women was associated with a lower likelihood of being persistently active OR = 0.32 (95% CI: 0.15, 0.69), *P* < 0.01. In men being married/living with a partner compared with being single was associated with becoming inactive, OR = 3.23 (95% CI: 1.47, 7.10), *P* < 0.01. In women, but not men, pain was significantly associated with a lower odds of remaining active, OR = 0.65 (95% CI: 0.49,0.86). Lastly in women, but not men, alcohol intake was significantly associated with becoming active, OR = 2.22 (95% CI: 1.10,4.50), *P* < 0.05, and conversely hypertensive status was associated with a diminished likelihood of becoming active, OR = 0.38 (95% CI: 0.18, 0.83), *P* < 0.05).

### Age-stratified analyses

#### HCHS/SOL

In older adults (> = 50 years), increasing age was more strongly associated with a lower likelihood of remaining persistently active, OR = 0.3 (95% CI: 0.18, 0.52), compared to younger adults, OR = 0.61 (95% CI: 0.43, 0.83). But in younger adults, being male was more strongly associated with remaining persistently active, OR = 4.23 (95% CI: 2.2, 8.14), compared to older adults, OR = 2.66 (95% CI: 1.58, 4.48). **(**See Additional Table 4a.) Higher household income was associated with a lower likelihood of remaining persistently active in older participants, OR = 0.64 (95% CI: 0.45, 0.9). There was also a suggestive association between remaining persistently active in the < 50 yr group who have hypertension OR = 2.05 (95% CI: 1.06, 3.98), *P* < 0.05. Interestingly younger, but not older participants who experienced an increase in work hours over the two time points had a suggestive higher odds of becoming active vs. remaining inactive OR = 1.99 (95% CI: 1.06, 3.71), *P* < 0.05.

#### FHS

In contrast to HCHS/SOL, household income among FHS participants was associated with higher odds of remaining persistently active in younger, but not in older groups although the significance level did not reach our *P* < 0.01 threshold of significance, OR = 1.53 (95% CI: 0.99, 2.35), *P* = 0.05 (see Additional Table 4b). Greater than high school education was associated with higher odds of being persistently active in older age group. In contrast to HCHS/SOL, among members of the FHS cohort, higher scores on the SF12- General Health showed an association suggesting higher likelihood of remaining persistently active in both the older age group, OR: 1.52 (95% CI: 1.07, 2.15) and in the younger age group, OR = 1.43 (95% CI: 1.11, 1.83). In contrast to HCHS/SOL participants, in FHS participants higher scores on SF-12 pain was associated with a lower odds of remaining active in older group, OR = 0.59 (95% CI: 0.42,0.83) and also tended to be associated with a higher odds of becoming inactive in younger group, OR = 1.35 (95% CI: 1.01,1.79). It is noteworthy that in the younger, but not older group, odds of becoming active vs. remaining inactive over time were somewhat higher among participants married/living with a partner vs. being single OR = 2.47 (95% CI: 1.18,5.15), *P* < 0.05.

## Discussion

Across Hispanic/Latino and non-Hispanic/Latino cohorts that were studied in a similar manner, we identified both shared and distinct predictors of change over time in MVPA. In an analysis of both Hispanic/Latino and non-Hispanic/Latino US adults age, sex, BMI, and diet quality were associated similarly with physical activity changes in an approximately eight-year period of follow-up. In both FHS and HCHS/SOL, more men than women, younger than older individuals, and individuals with lower BMI and higher diet quality were significantly more likely to be physically active at both assessments. However, variables such as income, education, marital status, depression, and general perception of health and pain, were associated differently with changes in physical activity in the two cohorts.

In older HCHS/SOL participants, higher income was associated with a greater likelihood of becoming inactive at the second assessment. In younger FHS participants, by contrast, higher income was associated with being persistently active across time. FHS consisted of primarily suburban White participants with sedentary occupations who seemed to derive their physical activity from leisure time which was likely higher with higher income and education. In contrast, HCHS/SOL participants resided mostly in urban communities, and approximately half of working individuals had occupations requiring a moderate to a high level of physical activity [9, 22, 29]. Having higher incomes in younger FHS participants was associated with a higher odds of remaining active. In FHS, higher levels of education were associated with remaining active at both time points. In contrast, education was not associated with changes in physical activity in HCHS/SOL.

For HCHS/SOL women, but not FHS women, greater prevalence of symptoms of depression was associated with becoming active at the second visit. Similar findings related to being employed, Spanish speaking, and being active have been reported in another study using the 2003–2006 National Health and Nutrition Examination Survey (NHANES) [30]. Interestingly being male and married, but not being female and married was associated with becoming inactive in both cohorts. Among FHS participants, higher SF-12 General Health scores, which is the perception of one’s general health status, were also associated with remaining active at both assessments. However pain was associated with higher odds of remaining inactive in FHS, but not in HCHS/SOL participants. These contrasting findings between the Hispanic/Latino and non-Hispanic/Latino populations suggest important differences in the life circumstances associated with higher physical activity. Among Hispanics/Latino adults, there may be a tendency for physical activity to correlate with hardships and demands that are accepted despite the pain and associated with family, work, or other responsibilities. In non-Hispanic/Latino adults, who participate in less labor-intensive job classes [9] an observable correlation between high physical activity levels and greater self-reported health emerged; possibly this indicates that in the non-Hispanic/Latino population, a regular exercise routine tends to be part of one’s self-perception of being in good health.

Strengths of this investigation include two large well-characterized cohorts of Hispanic/Latino and non-Hispanic/Latino adults of approximately similar age with two physical activity assessments collected near the same time period and using the same accelerometer. Limitations relate to activities that may not be captured by the Actical, such as bicycling and activities related to the upper body such as strength training, lifting, and other movements limited to the arms and shoulders. Other limitations are that FHS participants were primarily White participants of European descent, limiting the generalizability of results. The HCHS/SOL participants, while from four different cities in the US, do not represent US Hispanics/Latinos living in rural areas nor those living in urban and suburban regions that do not have the character of Hispanic/Latino enclaves. With respect to the socioecological model, this study focused on intrapersonal level measures. Future studies should incorporate other levels of the model including interpersonal, cultural, and environmental levels. Lastly about 80% of HCHS/SOL participants were foreign-born, while the percentage of foreign-born Latinos in the US who are 18 yr or older is about 45% which may limit generalizability [31].

## Conclusion

We observed associations between socio-demographic and biobehavioral variables and MVPA over time which may indicate different targets for intervention by age and gender and highlight the importance of understanding the context of the population being studied especially with respect to education, income, marital status, depressive symptomology and perception of health and pain. In summary, using targeted strategies to identify individuals in most need of support for physical activity will help achieve the 2018 Physical Activity Guidelines for Americans [14].

### Supplementary Information

**Additional file 1.**

**Additional file 2.**

## Data Availability

The Biologic Specimen and Data Repository Information Coordinating Center (BioLINCC) serves as the data repository center for HCHS/SOL: //biolincc.nhlbi.nih.gov/home.

## Abbreviations

HCHS/SOL: Hispanic Community Health Study/Study of Latinos
FHS: Framingham Heart Study
BMI: Body Mass Index
HbA1c: Hemoglobin A1c
FBG: Fasting Blood Glucose
LPA: Light Physical Activity
MVPA: Moderate-to-Vigorous Physical Activity
PA: Physical activity
V1: Visit 1
V2: Visit 2

## References

[1] Paffenbarger RS, Hyde RT, Wing AL, Hsieh CC (1986). Physical activity, all-cause mortality, and longevity of college alumni. N Engl J Med.

[2] Lieberman DE, Kistner TM, Richard D, Lee IM, Baggish AL. The active grandparent hypothesis: Physical activity and the evolution of extended human healthspans and lifespans. Proc Natl Acad Sci U S A. 2021;118(50):e2107621118. 10.1073/pnas.2107621118 PMC8685690 34810239

[3] Cuthbertson CC, Moore CC, Sotres-Alvarez D, Heiss G, Isasi CR, Mossavar-Rahmani Y, Carlson JA, Gallo LC, Llabre MM, Garcia-Bedoya OL (2022). Associations of steps per day and step intensity with the risk of diabetes: the Hispanic Community Health Study / Study of Latinos (HCHS/SOL). Int J Behav Nutr Phys Act.

[4] Schneiderman N, Llabre M, Cowie CC, Barnhart J, Carnethon M, Gallo LC, Giachello AL, Heiss G, Kaplan RC, LaVange LM (2014). Prevalence of diabetes among Hispanics/Latinos from diverse backgrounds: the Hispanic Community Health Study/Study of Latinos (HCHS/SOL). Diabetes Care.

[5] Cordero C, Schneiderman N, Llabre MM, Teng Y, Daviglus ML, Cowie CC, Cai J, Talavera GA, Gallo LC, Kaplan RC (2022). Diabetes Incidence Among Hispanic/Latino Adults in the Hispanic Community Health Study/Study of Latinos (HCHS/SOL). Diabetes Care.

[6] Colberg SR, Sigal RJ, Yardley JE, Riddell MC, Dunstan DW, Dempsey PC, Horton ES, Castorino K, Tate DF (2016). Physical Activity/Exercise and Diabetes: A Position Statement of the American Diabetes Association. Diabetes Care.

[7] Chen GC, Qi Q, Hua S, Moon JY, Spartano NL, Vasan RS, Sotres-Alvarez D, Castaneda SF, Evenson KR, Perreira KM (2020). Accelerometer-assessed physical activity and incident diabetes in a population covering the adult life span: the Hispanic Community Health Study/Study of Latinos. Am J Clin Nutr.

[8] Mossavar-Rahmani Y, Hua S, Qi Q, Strizich G, Sotres-Alvarez D, Talavera GA, Evenson KR, Gellman MD, Stoutenberg M, Castañeda SF (2020). Are sedentary behavior and physical activity independently associated with cardiometabolic benefits? The Hispanic Community Health Study/Study of Latinos. BMC Public Health.

[9] Kaplan RC, Song RJ, Lin J, Xanthakis V, Hua S, Chernofsky A, Evenson KR, Walker ME, Cuthbertson C, Murabito JM (2022). Predictors of incident diabetes in two populations: framingham heart study and hispanic community health study / study of latinos. BMC Public Health.

[10] Bauman AE, Reis RS, Sallis JF, Wells JC, Loos RJ, Martin BW (2012). Correlates of physical activity: why are some people physically active and others not?. Lancet.

[11] Kompf J, Rhodes R. Differential correlates for aerobic physical activity and resistance training: a systematic review. Psychol Health Med. 2022:1–21. 10.1080/13548506.2022.2142617 36373398

[12] Evans JT, Phan H, Buscot MJ, Gall S, Cleland V (2022). Correlates and determinants of transport-related physical activity among adults: an interdisciplinary systematic review. BMC Public Health.

[13] Trost SG, Owen N, Bauman AE, Sallis JF, Brown W (2002). Correlates of adults' participation in physical activity: review and update. Med Sci Sports Exerc.

[14] Physical Activity Guidelines for Americans [https://health.gov/sites/default/files/2019-09/Physical_Activity_Guidelines_2nd_edition.pdf].

[15] Sorlie PD, Avilés-Santa LM, Wassertheil-Smoller S, Kaplan RC, Daviglus ML, Giachello AL, Schneiderman N, Raij L, Talavera G, Allison M (2010). Design and implementation of the Hispanic Community Health Study/Study of Latinos. Ann Epidemiol.

[16] Lavange LM, Kalsbeek WD, Sorlie PD, Avilés-Santa LM, Kaplan RC, Barnhart J, Liu K, Giachello A, Lee DJ, Ryan J (2010). Sample design and cohort selection in the Hispanic Community Health Study/Study of Latinos. Ann Epidemiol.

[17] Gallo LC, Carlson JA, Sotres-Alvarez D, Sallis JF, Jankowska MM, Roesch SC, Gonzalez F, Geremia CM, Talavera GA, Rodriguez TM (2019). The Hispanic Community Health Study/Study of Latinos Community and Surrounding Areas Study: sample, design, and procedures. Ann Epidemiol.

[18] Gallo LC, Carlson JA, Sotres-Alvarez D, Sallis JF, Jankowska MM, Roesch SC, Gonzalez F, Geremia CM, Talavera GA, Rodriguez TM (2019). Corrigendum to 'The Hispanic Community Health Study/Study of Latinos Community and Surrounding Areas Study: sample, design, and procedures' Annals of Epidemiology Volume 30, (2019) 57–65. Ann Epidemiol.

[19] Dawber TR, Meadors GF, Moore FE (1951). Epidemiological approaches to heart disease: the Framingham Study. Am J Public Health Nations Health.

[20] Splansky GL, Corey D, Yang Q, Atwood LD, Cupples LA, Benjamin EJ, D'Agostino RB, Fox CS, Larson MG, Murabito JM (2007). The Third Generation Cohort of the National Heart, Lung, and Blood Institute's Framingham Heart Study: design, recruitment, and initial examination. Am J Epidemiol.

[21] Spartano NL, Wang R, Yang Q, Chernofsky A, Murabito JM, Vasan RS, Levy D, Beiser AS, Seshadri S (2023). Association of accelerometer-measured physical activity and sedentary time with epigenetic markers of aging. Med Sci Sports Exerc..

[22] Arredondo EM, Sotres-Alvarez D, Stoutenberg M, Davis SM, Crespo NC, Carnethon MR, Castañeda SF, Isasi CR, Espinoza RA, Daviglus ML (2016). Physical Activity Levels in U.S. Latino/Hispanic Adults: Results From the Hispanic Community Health Study/Study of Latinos. Am J Prev Med.

[23] Evenson KR, Sotres-Alvarez D, Deng YU, Marshall SJ, Isasi CR, Esliger DW, Davis S (2015). Accelerometer adherence and performance in a cohort study of US Hispanic adults. Med Sci Sports Exerc.

[24] Choi L, Liu Z, Matthews CE, Buchowski MS (2011). Validation of accelerometer wear and nonwear time classification algorithm. Med Sci Sports Exerc.

[25] Colley RC, Tremblay MS (2011). Moderate and vigorous physical activity intensity cut-points for the Actical accelerometer. J Sports Sci.

[26] Williams MW, Li CY, Hay CC (2020). Validation of the 10-item center for epidemiologic studies depression scale post stroke. J Stroke Cerebrovasc Dis.

[27] Li Y, Mirzaei F, O'Reilly EJ, Winkelman J, Malhotra A, Okereke OI, Ascherio A, Gao X (2012). Prospective study of restless legs syndrome and risk of depression in women. Am J Epidemiol.

[28] Blank K, Gruman C, Robison JT (2004). Case-finding for depression in elderly people: balancing ease of administration with validity in varied treatment settings. J Gerontol A Biol Sci Med Sci.

[29] Singer RH, Stoutenberg M, Gellman MD, Archer E, Davis SM, Gotman N, Marquez DX, Buelna C, Deng Y, Hosgood HD (2016). Occupational physical activity and body mass index: results from the Hispanic Community Health Study/Study of Latinos. PLoS ONE.

[30] Jones SA, Wen F, Herring AH, Evenson KR (2016). Correlates of US adult physical activity and sedentary behavior patterns. J Sci Med Sport.

[31] Latinos see US as better than place of family’s ancestry for opportunity, raising kids, health care access. [https://www.pewresearch.org/race-ethnicity/2022/01/20/latinos-see-u-s-as-better-than-place-of-familys-ancestry-for-opportunity-raising-kids-health-care-access/].

