# Supplementary material for: Characterizing longitudinal change in accelerometry-based moderate-to-vigorous physical activity in the Hispanic Community Health Study/Study of Latinos and the Framingham Heart Study
Source: BMC Public Health. 2023 Aug 24;23:1614. doi: 10.1186/s12889-023-16442-9 (PMC10464120; doi:10.1186/s12889-023-16442-9)
Supplement: Supplementary file 2 — Additional file 2. [file 12889_2023_16442_MOESM2_ESM.docx]

**HCHS/SOL and FHS: Variable Definitions**

**HCHS/SOL**

- First visit is comprised of N=16,415 (age 18-74) and N=11,623 in the second visit
- First visit is 2008-2011 and second visit is 2017-2019
- Participants with acceptable physical activity data from both visits (N=3823).
- Participants with CVD at baseline were excluded from multivariate regression analysis. The final sample size is n=3646 for regression analyses.

**FHS**

- Gen 3 (N=4095) plus Omni 2 (N=410) and NOS (N=103)
- Of those, N=3269 participated in exam cycle 2 (first visit) and returned for exam cycle 3

(Gen 3 n=2940, Omni 2 n=278, NOS n=51)

- - We exclude those without physical activity measures in either exam 2 and/or 3 to get a sample size of N=2009 (Gen 3 n=1825, Omni 2 n=156, NOS n=28)
- Exam cycle 2 is 2008-2011 and cycle 3 is 2016-2019

**Physical Activity Outcome Variable Definition**

- MVPA guideline per 2018 Physical Activity - more than 150 minutes of moderate-intensity activity a week (or more than 75 minutes of vigorous-intensity physical activity a week) or the equivalent combination of moderate and vigorous activity. All activity measures except MVPA were adjusted by daily wear time and standardized to a 16 hour wear day.

**Selected Baseline characteristics/Covariates Definition**

- Change in work hour
  - Decreasing: Full-time 🡪 part-time/not employed/retired, Part-time 🡪 not employed/retired, not employed 🡪 retired
  - Stable: no change
  - Increasing: part-time/not employed/retired 🡪 full-time, not employed/retired 🡪 part-time, retired 🡪 not employed
- Prevalent CVD

HCHS/SOL definition:

- Prevalent CVD at baseline (had heart attack, had a balloon angioplasty, a stent, or bypass surgery to the arteries in your heart to improve the blood flow to your heart or had stroke).

FHS definition:

- Fatal or nonfatal coronary heart disease (includes angina, acute coronary syndrome/coronary insufficiency, myocardial infarction), cerebrovascular event (stroke or TIA), overt heart failure, and peripheral arterial disease.
- Diabetes/Prediabetes

HCHS/SOL definition:

- - If (fasting time > 8 hr AND fasting glucose in range 100-125 mg/dL) or (post-OGTT glucose in range 140-199 mg/dL) or (5.7%≤A1C these participants are categorized as prediabetic
  - If (fasting time > 8 hr AND fasting glucose ≥126 mg/dL) or (fasting time ≤8 hr AND fasting glucose ≥200 mg/dL) or (post-OGTT glucose ≥200 mg/dL) or (A1C ≥6.5%) or self-report of diabetes, then these participants are categorized as diabetic

FHS definition**:**

- - If 100 mg/dL ≤ Fasting Blood Glucose < 126 mg/dL and HbA1c ≤ 6.5 -*or*- 5.7 ≤ HbA1c ≤ 6.5 and FBG < 126 mg/dL, participants are categorized as pre-diabetic
  - If HbA1c > 6.5 then these participants are categorized as diabetic
- SF12 General Health
  - In general, would you say your health is: 0= Poor,1= Fair,2=Good,3= Very good,4= Excellent
- Depressive Symptomology
  - Defined as CESD-10 score ≥10 in HCHS/SOL
  - Defined as CESD-20 score ≥ 16 in FHS

**Multinomial Logit Models**

In the following analyses, we performed multinomial logit models on a physical activity outcome with 4 levels. The outcome levels are determined by the change in physical activity, as measured by MVPA guidelines. Models are adjusted by age and sex (Model 0), socio-demographics (Models 1 and 2), medical history (Model 3), and medications (Model 4). Stratified analysis were performed by gender and age (<50, ≥50 years old) for model 3 only.

| **At Baseline** | **At Follow-Up** | **Outcome Category** |
| --- | --- | --- |
| MVPA Guideline Not Met | MVPA Guideline Not Met | Remained Inactive (Reference) |
| MVPA Guideline Not Met | MVPA Guideline Met | Became Active |
| MVPA Guideline Met | MVPA Guideline Not Met | Became Inactive |
| MVPA Guideline Met | MVPA Guideline Met | Remained Active |
